# Integrating Science on *Xanthomonas* and *Xylella* for Integrated Plant Disease Management

**DOI:** 10.3390/microorganisms11010006

**Published:** 2022-12-20

**Authors:** Joana Costa, Joël F. Pothier, Jens Boch, Emilio Stefani, Ralf Koebnik

**Affiliations:** 1 University of Coimbra, Centre for Functional Ecology, Department of Life Sciences, 3000-456 Coimbra, Portugal; 2 Laboratory for Phytopathology, Instituto Pedro Nunes, 3030-199 Coimbra, Portugal; 3 Environmental Genomics and Systems Biology Research Group, Institute of Natural Resource Sciences, Zurich University of Applied Sciences (ZHAW), 8820 Wädenswil, Switzerland; 4 Institute of Plant Genetics, Leibniz Universität Hannover, 30419 Hannover, Germany; 5 Department of Life Sciences, University of Modena and Reggio Emilia, 42122 Reggio Emilia, Italy; 6 Plant Health Institute of Montpellier, University of Montpellier, CIRAD, INRAE, Institut Agro, IRD, 34394 Montpellier, France

## 1. Prologue

Present, emerging or re-emerging plant diseases due to infection by bacteria of the *Lysobacteraceae* (syn: *Xanthomonadaceae*) family are continually challenging food security and cause significant losses to the economies of European countries each year. To address this challenge, concerted R&D actions at the international level were supported by the COST Action networking instruments. COST (European Cooperation in Science and Technology) is a pan-European intergovernmental framework with the mission of enabling break-through scientific and technological developments leading to new concepts and products, thereby contributing to strengthening Europe’s research and innovation capacities.

The ‘EuroXanth’ COST Action (https://euroxanth.eu, accessed on 28 November 2022), which ran from spring 2017 to autumn 2021, addressed several key aspects of the pathogen–vector–host interactions from the cellular to the population level, such as the population structures and virulence mechanisms of the pathogens, molecular mechanisms underlying disease resistance to the pathogens, proof-of-concepts for the development of durably resistant plant cultivars and bio-control schemes tailored to population and pathogen. 

‘EuroXanth’ COST Action generated a pan-European platform that gathered experts from different disciplines, such as molecular diagnostics, molecular host–microbe interactions, plant resistance breeding and applied microbiology. To develop and implement effective plant protection schemes, be it via resistant crop cultivars or other control mechanisms, four working groups were implemented: (i) Diagnostics and Diversity—Population Structure; (ii) Pathogen Biology; (iii) Genetic Resistance—Host Defence; and (iv) Disease Management—Vector Control. Here, the four working group leaders and the chair of COST Action edited a series of manuscripts that are believed to help achieve the COST Action’s goals.

## 2. Integrating Science on *Xanthomonas* and *Xylella* for Integrated Plant Disease Management

### 2.1. Diagnostics and Diversity—Population Structure

The bacteria of the genus *Xanthomonas* can infect a panoply of crops and wild plants responsible for diseases with severe economic and environmental impacts on seed, plant and food trade. Identification of the causative agents is quite complex, since infections present a wide range of non-specific symptoms and the co-existence of phylogenetically close strains with distinct phenotypes; this poses an additional diagnostic challenge. Data on future climate scenarios predict an increase in the severity and frequency of epidemics, as well as a geographical spread of pathogens, with consequent increased pressure on phytosanitary services. In this context, the effectiveness of integrated disease management strategies is strongly conditioned by the availability of rapid, sensitive and specific diagnostic methods. In recent years, the accumulation of genomic data has facilitated the development of more sensitive and specific methods. Within this framework, the review from Catara et al. [1] discussed changes in the phytosanitary status of regulated *Xanthomonas* in the European Union and the consequences for the official controls. The authors contextualised the scope, relevance and constraints associated with the development and implementation of official diagnostic protocols for regulated *Xanthomonas* and compiled a comprehensive list of diseases, regulations and recommendations for their management. They also reviewed the scope, limitations and applications of molecular methods based on specific DNA targets for the detection, diagnosis, identification and molecular typing of regulated xanthomonads. State-of-the-art applications and limitations of genetics-informed diagnosis protocols were discussed by the authors, ending the review with a reflection on the main pitfalls, trends and future challenges to the detection, identification and diversity studies of plant-pathogenic xanthomonads. 

‘EuroXanth’ COST Action has seen its name immortalised after a new pathogen affecting walnut production was named after it, called *Xanthomonas euroxanthea*. Silva et al. [2] developed DNA markers for the molecular detection of this new pathogen. Of the eight markers identified, the authors found that four were located in highly syntenic regions across eleven *X*. *euroxanthea* genomes. Three of these four DNA markers were combined in a multiplex PCR, allowing the confident identification of *X*. *euroxanthea* strains with a detection limit of 1 ng per PCR reaction. Three markers totalising 1180 bp were also reported to have an equal discriminatory power as the usual partial housekeeping sequences covering 2774 bp, thus, underlining their typing potential for *X*. *euroxanthea* lineages. 

*Xanthomonas gardneri*, since then taxonomically revised as *X. hortorum* pv. *gardneri*, is responsible for the bacterial spot, an economically important disease for tomato and pepper production. To improve the detection of this bacterium, Stehlíková et al. [3] developed and optimised a new LAMP-based real-time monitoring method. Data on the specificity and sensitivity of the assay were provided based on results obtained with the set of bacterial strains pathogenic for tomato and pepper and from infected plants. The authors further discussed the advantages, limitations and applications of this assay in the context of disease management and control, in the complex context of diseases caused by other *Xanthomonas* spp. 

In addition to *X. hortorum* pv. *gardneri*, two pathovars of *Xanthomonas euvesicatoria* cause bacterial spots on pepper and tomato plants. In order to trace back the spread of bacterial spot pathogens within and among countries, Vancheva et al. [4] developed the first multilocus variable number of tandem repeat analyses (MLVA) scheme for pepper- and tomato-pathogenic strains of *X. euvesicatoria* and applied this new tool to analyse a representative set of 88 *X. euvesicatoria* pepper strains from Bulgaria and North Macedonia. Strains from the different regions of these countries were found to be widespread in genetically distant clonal complexes or singletons, arguing for the long-term coexistence of different populations and several introduction events in this area of the Balkan Peninsula. 

Expansion to new geographic areas is a key feature of emerging and invasive plant pests, and islands play a specific role in this as they are surrounded by natural borders. Nevertheless, pathogens often travel together with their host plant or plant products along economic markets to cross borders. Pruvost et al. [5] analysed the diversity of the bacterial citrus pathogen *Xanthomonas citri* pv. *citri* in a collection of more than 500 strains sampled from citrus plants on several islands in the South-West Indian Ocean (SWIO) region. There is a high prevalence of the citrus canker disease caused by *X. citri* pv. *citri* on these islands, causing frequent outbreaks. Using genotyping and pathogenicity tests, the authors identified the worldwide prevalent pathotype A genetic lineage 1 as predominant on the islands, but the strains displayed marked genetic differences, indicating several independent introduction events on the different islands and regions on the islands. Furthermore, for the first time, this study identified genetic lineage 4 pathotype A* strains on three islands of the SWIO region. Interestingly, the movement of strains between islands, e.g., via contaminated citrus plants, was very limited. Within an island, the distribution of strains differed, suggesting that infected plants from commercial nurseries might play an important role in the spread of the pathogen. 

Complementary to micro- and minisatellites, as examined in MLVA typing, CRISPR arrays represent another class of rapidly evolving genetic loci that are useful for molecular typing, diversity studies and global epidemiological surveillance. Bellanger et al. [6] updated previous work on the CRISPR typing of *X. citri* pv. *citri* by analysing a set of 355 strains for which genomic information was available. They doubled the number of known absence/presence patterns of CRISPR spacers in this pathovar, also called spoligotypes (spacer oligotypes), and proposed parsimonious models for their evolutionary trajectory in all three pathotypes of *X. citri* pv. *citri*. Notably, it was even possible to reconstruct the spoligotype of an ancient DNA sample retrieved from an herbarium specimen, thus, allowing such an old representative of the pathovar to be placed in the context of modern isolates. The authors continued to analyse the remaining ~30 pathovars of *X. citri*, thus, revealing the presence of the oldest part of the CRISPR array in the ancestor of several pathovars of *X. citri*. Altogether, this study generated a useful framework for further analyses of CRISPR loci and gave hints about the global spread of the citrus canker pathogen, as exemplified by at least two introductions in West Africa. 

### 2.2. Pathogen Biology

*Xylella fastidiosa* is a member of the *Lysobacteraceae* family capable of colonising the xylem of more than 500 host plant species, as well as xylem-feeding insects belonging to the *Cicadellinae* (sharpshooters) and *Cercopoidea* (spittlebugs). It is the causal agent of diseases with significant economic impact, representing a devastating phytosanitary emergency that severely affects numerous agroforestry systems. To date, knowledge about the molecular mechanisms underlying its pathogenicity is limited, as well as the characterization of its phenotypic and biological traits. D’Attoma et al. [7] investigated in vitro the behaviour of the *X. fastidiosa* De Donno strain—which is responsible for the rapid olive decline syndrome discovered in 2013 in olive groves in southern Italy—and compared its relevant biological traits with those of the Temecula1 strain, which was isolated from a naturally infected grapevine with Pierce’s disease in California. They demonstrated that the De Donno strain showed no fringing on the agar plates, produced larger amounts of biofilm, and had more aggregative behaviour than the Temecula1 strain. In addition, based on a computational analysis, potentially deleterious sequence variations were identified that most likely affect natural competence and the lack of fringe formation. GFP-producing and *rpfF* mutant strains were obtained by co-electroporation, allowing the future exploration of pathogen–host and pathogen–insect vector interactions.

Fernandes et al. [8] determined three complete genome sequences of *X*. *euroxanthea* and one of *Xanthomonas arboricola* pv. *juglandis* from isolates of a single walnut tree in Portugal. These included two walnut-pathogenic and two non-pathogenic strains. Their comparative genomic analyses between the two species revealed remarkable genomic differences, which translated into different pathogenicity and virulence features. This concerned the type 3 secretion system (T3SS) and its effectors and other secretory systems, chemotaxis-related proteins, and extracellular enzymes. Where a pattern of homologous genes was observed—which are thought to be associated with pathogenicity, virulence, and niche-specific adaptations—the authors concluded that these need to be investigated in future functional studies to determine their importance in walnut diseases caused by *Xanthomonas*. 

Stewart and Ronald [9] used the potential of comparative genomics to infer likely gene functions for three different two-component regulatory systems, RaxH-RaxR, VgrS-VgrR, and DetS-DetR, which have been denoted as ColS-ColR in various genome annotations and publications. They found that the *Xanthomonas* RaxH-RaxR system (Required for AvrXa21 activity) is orthologous to the *Pseudomonas* ColS-ColR, which regulates lipid A remodelling, whereas the other two systems are unrelated to the Col system. Notably, three genes adjacent to *raxRH* were predicted to encode enzymes that are thought to remodel the lipid A component of lipopolysaccharide, and corresponding modifications had indeed been previously detected in lipid A from multiple *Xanthomonas* species. 

Uceda-Campos et al. [10] used comparative genomics of almost 100 whole-genome sequences to find potential candidates related to host specificity in the species *X. fastidiosa*. However, using a score of mapping metrics that estimates the clade depth where organisms share a trait, as applied to phylogenetic trees for 1605 orthologous CDSs, no candidate host-specificity determinants were strongly supported. The authors then explored the accessory genome, which they found to be represented by an abundant and heterogeneous mobilome, including a diversity of prophage regions. Altogether, these contributions demonstrate the power and limitations of comparative genomics in formulating new hypotheses, which may then be addressed by experimental approaches. 

### 2.3. Genetic Resistance—Host Defence

Gétaz et al. [11] investigated the host–pathogen interactions between the strawberry pathogen *Xanthomonas fragariae* and its host *Fragaria* × *ananassa* using whole transcriptome sequencing. Their dual RNA-seq analysis used two time points to compare the pathogen and host response between the stages of early visible (12 days post-inoculation [dpi]) and well-developed symptoms (29 dpi). They found a total of 361 plant and 144 bacterial genes that were significantly differentially expressed. Among the higher expressed genes in the plant, they identified pathogen-associated molecular pattern (PAMP) receptors and pathogenesis-related thaumatin-encoding genes. This study revealed that the strawberry plant changed gene expression to consistently adapt its metabolism to the progression of the infection. 

*X. citri* pv. *glycines* is a major pathogen of soybean in Korea, and Kang et al. [12] used comparative genomics to analyse pathogenicity genes in five Korean strains and one strain from the USA. The authors observed that the six strains varied in number and diversity of candidate T3SS effector genes, with the five Korean strains being similar to each other but distinct from the US strain. In addition, their study revealed diverse repeat arrays in the transcription activator-like effectors (TALEs) among the six strains, falling into six groups based on their repeat variable di-residue (RVD) patterns and likely binding specificities in the host genomes. Notably, blocks of the repeat arrays were found to be shared among several TALEs, suggesting that recombination and domain swapping had occurred in *X. citri* pv. *glycines* during the evolution of the TALE genes, thus, perhaps evolving the target specificity of the DNA-binding domains in soybean cultivars. 

*Xanthomonas phaseoli* pv. *manihotis* causes bacterial blight disease on cassava plants, which is a severe and widespread disease not only in Africa, but also in South America. Zárate-Chavez et al. [13] focused on TALEs as a key virulence factor of *X. phaseoli* pv. *manihotis* to characterise the diversity of pathogenic strains and the adaptations in their virulence repertoire throughout Colombia. TALEs induce the expression of specific plant genes based on their central repetitive DNA-binding domain. These repeats are particularly prone to recombination and rearrangements to evolve novel DNA-binding specificities and adapt to novel targets. At the same time, it is possible to predict the DNA-binding specificity of TALEs and, thereby, identify their plant targets. The authors characterised the TALEs in the Colombian strains and identified novel repeat compositions, leading them to propose an evolutionary diversification from possible common TALE ancestor genes. Furthermore, they identified host genes that are induced by two *X. phaseoli* pv. *manihotis* strains, among them a previously known virulence target (*MeSWEET10a*, a sucrose transporter) and characterised potential novel targets, which are a good starting point to better understand how *X. phaseoli* pv. *manihotis* colonises cassava plants. 

Citrus cankers caused by *X. citri* pv. *citri* and *X. citri* pv. *aurantifolii* are a serious threat to citrus cultivation worldwide but are not currently found in Europe and the Mediterranean Basin. Often, pathogens are not purely restricted to one host plant, but may colonize related or even unrelated plants, thereby exhibiting a complex host–pathogen network. Both *X. citri* pathovars have different pathogenic variants (pathotypes) with different host ranges. To understand the possible risks of introducing *X. citri* pathogens, Licciardello et al. [14] studied the susceptibility to these pathogens not only of classical citrus crop plants, but also of a diversity of ornamental plants from the *Rutaceae* family. The authors applied a thorough experimental approach testing 32 plant accessions against seven strains from different *X. citri* pathotypes to obtain a much broader picture of possible host plants than existed before. Together with other studies, this work showed that *X. citri* could infect a large number of plants in the *Rutaceae* family, calling for careful quarantine management in international trade including ornamental plants. 

### 2.4. Disease Management—Vector Control

The management of the several diseases caused by xanthomonads is particularly cumbersome due to the lack of systemic bactericidal chemicals. Indeed, copper-based substances are, currently, the first choice of pesticides to be used, both in organic production and in conventional agricultural systems. Regretfully, several xanthomonads have evolved a pronounced resistance to copper that is based on the presence of specific gene sequences located on both plasmids and the chromosome. A possible ‘green’ solution to this challenge is presented by Stefani et al. [15], who provided indications on the application of bacteriophages in agriculture. Bacteriophages are viruses that infect bacteria and are present in any environment; the search, selection, identification and testing of specific bacteriophages may lead to the development of innovative biopesticides able to efficiently control phytopathogenic bacteria and, among them, copper-resistant xanthomonads. The paper of Stefani et al. also investigated the legal background and industrial property issues of bacteriophage-based biopesticides, e.g., the registration requirements, taking into consideration the present situation in the European Union and the United States of America. 

A strategy for the implementation of control methods requires a deep knowledge of how xanthomonads adapt to and persist in the agro-environment. To shed further light on the importance of lipopolysaccharides (LPS) and exopolysaccharides (EPS) in xanthomonads, Picchi et al. [16] mutated the *xanA* gene of *X. citri* pv. *citri*, a key gene for LPS and EPS biosynthesis. The mutant strain was found to differ in the saccharide composition, ultimately leading to a reduction in xanthan yield in the *xanA*-deficient strain, but also affecting other important features in *X. citri*, such as biofilm formation and sliding motility. Importantly, the *xanA*-deficient strain caused no symptoms on host leaves upon spray inoculation, a method that mimics the natural infection process. These results suggest that *xanA* plays an important role in the epiphytical stage of *X. citri* pv. *citri*, one of the most destructive xanthomonads, and is essential for successful interaction with the host plant. This finding opens new perspectives for the biocontrol of *X. citri* pv. *citri*, for instance, using a microbial biocontrol agent able to inhibit the formation of a biofilm by the pathogen. 

Citrus cankers caused by *X. citri* pv. *citri* cannot be cured and agricultural practices in citrus production are, therefore, important to control this disease. One particular threat to underestimate infections is the dormant or persister cells of the pathogen, which have a lowered metabolism and, thereby, an increased tolerance to antimicrobials that rely on active metabolic pathways. This phenomenon is well-known in human-pathogenic bacteria, but not well-understood in plant pathogens. Martins et al. [17] describe different environmental triggers that induce the formation of such persisters in *X. citri* pv. *citri*. Among these are antibiotics, high temperature, and copper or zinc metals. The authors further describe different chemicals that can activate and kill *X. citri* pv. *citri* persister cells. This work provides interesting suggestions for plant disease management practices to better control citrus canker disease. 

A pivotal eco-environmental aspect of *X. campestris* pv. *campestris*, the causal agent of the black rot of Crucifers, was described by Gazdik et al. [18]. They monitored the persistence of the bacterial inoculum in the cropped field, discovering that the pathogen is able to survive at least two years in cabbage fields. These results are now giving strong indications on how to manage crucifer fields from the agronomic point of view once a disease outbreak is recorded, especially related to crop rotation and proposed surveillance procedures. 

During the last ten years, *X. fastidiosa* has become the most threatening and troublesome phytopathogen in the Mediterranean basin, especially when it affects olive groves. As an alien pest of recent introduction, the disease caused by *X. fastidiosa* dramatically spread and resulted in the death of millions of trees. In their review, Morelli et al. [19] analysed all the efforts made by scientists, phytosanitary services, agronomists and growers to reduce the spread of the pathogen, based on the knowledge of its epidemiology, vector(s) biology, orchards management and legislative measures. Morelli et al. describe a ‘historical’ path from the initial legislative measures that confused most policymakers and the massive use of insecticides against the vector(s) to the improved and more sustainable agronomic practices currently applied in olive groves and the development of tolerant olive varieties. This path leads to the possibility of coexistence with *X. fastidiosa*, thus, making the future of olive cultivation in Italy and the Mediterranean basin less alarming than it was a few years ago.

## 3. Epilogue

This Special Issue on *Lysobacteraceae* highlights various aspects of plant-pathogenic xanthomonads, from diagnostics to disease management, that are of interest to different stakeholders concerned by plant diseases. Molecular epidemiology and insights into population structures are key to predicting and monitoring future outbreaks. Understanding the mechanisms of survival and persistence under unfavourable abiotic conditions and ways to counteract competing microbes may enable the development of new approaches to suppress pathogen load. Insights into signal transduction pathways, the ability to acquire and utilise nutrients and molecular mechanisms to suppress plant defences may lead to novel approaches to control plant-pathogenic bacteria. A better understanding of evolutionary pressures on pathogenic bacteria may allow better intervention in the emergence of new pathogenic lineages. In this direction, our manuscript collection will stimulate new research and, hopefully, contribute to an environmentally friendly and safe food supply at a reasonable cost.

## Data Availability

Not applicable.
